# Immunonutrition in Orthopedic and Traumatic Patients

**DOI:** 10.3390/nu15030537

**Published:** 2023-01-19

**Authors:** Pietro Gregori, Edoardo Franceschetti, Susanna Basciani, Lorenzo Impieri, Biagio Zampogna, Alfredo Matano, Carlo Manzi, Ludovico Carbone, Luigi Marano, Rocco Papalia

**Affiliations:** 1 Department of Orthopedic and Trauma Surgery, Campus Bio-Medico University, 00128 Rome, Italy; 2 Residency Program in Orthopedics and Traumatology, University of Milan, 20122 Milan, Italy; 3 Health Service Management Board, Azienda Ospedaliera di Caserta Sant’Anna e San Sebastiano, 81100 Caserta, Italy; 4 Health Service Management Board, Azienda Sanitaria Locale Caserta, 81100 Caserta, Italy; 5 Department of Medicine, Surgery and Neurosciences, University of Siena, 53100 Siena, Italy

**Keywords:** immunonutrition, orthopedics, nutrition, surgery

## Abstract

The role of nutrition intervention in surgical settings is constantly developing and evolving. Immunonutrition represents a viable option to reduce perioperative and postoperative complications in surgical oncology. However, as far as we know, little research has been conducted in the orthopedic field. With this review, we aim to summarize the state of the art in the application of immune-enhanced compounds to surgical, orthopedic, and traumatic patients. The PRISMA (Preferred Reporting Items for Systematic Reviews and Meta-Analyses) guidelines were adopted. A comprehensive search was carried out using the PubMed (MEDLINE), EMBASE, and Cochrane Library databases. All the studies dealing with immunonutrition fed to traumatic and orthopedic patients were pooled, the data were extracted, and the studies were discussed. A total of eight studies were included: six focused on trauma surgery and two on elective orthopedic surgery. The epidemiological characteristics of participants and the assessment of results were reported. Data were analyzed using R software (2020; R Core Team). Based on the current available literature, a positive impact of immunonutrition in orthopedic and trauma surgical settings was registered. All studies analyzed showed the favorable impact of the immunonutrition diet on clinical outcomes. The full effect of this type of nutrition and its different applications in the orthopedic and traumatic fields should be critically investigated through more extensive randomized controlled trials.

## 1. Introduction

Worldwide, traumatic injuries and osteoarthritis requiring elective surgery represent a significant and increasing challenge to healthcare systems [1], especially in older subjects [2,3]. Most traumatic injuries are orthopedic, with fragility fractures being the most common. Hip fractures are a priority fragility fracture due to short- and long-term complications leading to higher mortality, long-lasting functional limitations, and an increasing burden on healthcare services [2,4]. Moreover, elective hip and knee replacement arthroplasty is increasingly advocated by orthopedic surgeons for older adults with severe osteoarthritis to counteract pain and functional limitations. However, the risk of post-surgical morbidity may limit the access of eligible people and impair the efficacy of the surgical techniques even in the elective scenario, leading to several complications including, sarcopenia, functional decline, surgery revision, and additional healthcare burden [5].

Several conditions have been identified in the trauma and elective orthopedic settings to explain the failure of professionals’ and patients’ desired goals, with some authors raising age-related concerns over expected improvement and risks [6]. Poor nutrition status is a serious issue affecting more than 30% of hospitalized patients [7,8], reducing their quality of life, and hindering postoperative functional rehabilitation and fast discharge [9]. Interestingly, malnutrition is emerging as a significant factor in several surgical patient outcomes, leading to immune dysfunction, which can directly increase susceptibility to infection. Thus, the postoperative course strongly relates to nutrition, protein metabolism, and the prevention of surgical site or systemic infections [10]. 

Immune-enhancing nutrition may represent a powerful approach to help reduce all the above complications [11]. To date, preoperative administration of specific immune-enhancing supplements and so called “immunonutrition”, has been shown to improve wound healing and reduce postoperative complications, infectious diseases, and length of hospital stay in both surgical and oncological settings [12]. An immunonutrition diet, enriched with specific nutrients, which are mainly arginine, omega-3-fatty acids, glutamine, or ribonucleic acid, helps regulate the body’s response to illness and injury [13,14,15,16]. For many of these nutrients, an important role in modulating inflammation is reported in the literature: arginine is an essential amino acid during growth and wound healing; omega-3 fatty acids can modulate immunity and have an anti-inflammatory effect; and an RNA nucleotide-deficient diet results in lower IL-2 production and T-cell response [17,18,19]. Thus, nationally recognized guidelines support the use of immune-enhancing nutrition in patients with a high risk of adverse events. Previous studies have emphasized the use of immuno-nutrients in patients undergoing gastrointestinal surgery, mainly in the peri-operative period [12,20,21]. The administration of these compounds has been proven to be associated with lower infectious complications and length of hospital stay compared to standard nutrition, which, ultimately, greatly reduces the medical expenditure as well as improves the prognosis of the given patients [12,22,23,24,25]. Although randomized clinical trials have been conducted in urology [26], gynecology [27], and traumatic patients [10,12,17,28,29,30,31] showing similar findings, strong evidence in orthopedic settings is still lacking. 

Moreover, the perioperative enhanced recovery after surgery (ERAS) program includes nutritional supplements that can minimize the surgical stress response and morbidity rate and accelerate functional recovery, particularly in older persons, whose functional reserve is limited [32,33]. A shorter length of hospital stay is the main outcome achieved by ERAS, while the effects on physical performance remain to be investigated [32].

There is a lack of evidence about the effect of multimodal protocols, including immunonutrition, on perioperative complications and functional recovery in orthopedic surgery [34]. Thus, in light of such evidence, the present review aims at delineating the state-of-the-art in the application and benefits of perioperative immunonutrition protocols in orthopedic surgery.

## 2. Materials and Methods

**Figure 1:**
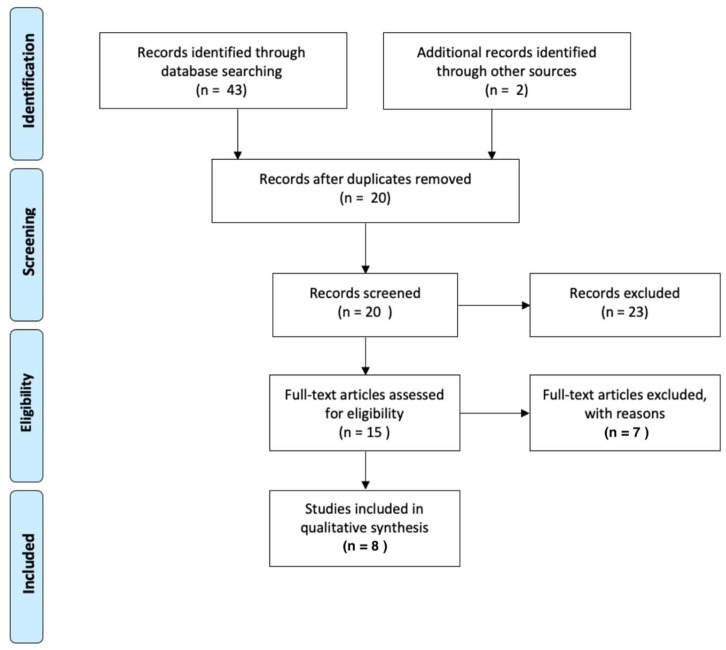
PRISMA (Preferred Reporting Items for Systematic Reviews and Meta-Analyses).

The review was conducted following PRISMA (Preferred Reporting Items for Systematic Reviews and Meta-Analyses) guidelines [35] (Figure 1).

### 2.1. Eligibility Criteria

Studies written in English, Italian, French, Spanish, and the German language were eligible. Only peer-reviewed journals were considered. Specifically, we included laboratory in vivo or in vitro studies, randomized controlled trials (RCTs), prospective and retrospective comparative studies, and case series, all dealing with the use of immunonutrition for orthopedic or traumatic patients. All studies focused on the use of immunonutrition for orthopedic or trauma patients. Exclusion criteria were reviews of the literature, expert opinions, and studies that did not evaluate the use of immunonutrition for orthopedic or trauma patients.

### 2.2. Information Sources and Search

A systematic electronic search of CINAHL, EMBASE, PubMed, and the Cochrane Central Registry of Controlled Trials was carried out by two reviewers (P.G. and L.M.), with the goal of identifying eligible studies published until May 2022. The utilized search strings were: ((Immunonutrition [MeSH Terms]) AND TraumaMeSH Terms]); (((Trauma [MeSH Terms]) AND Immunonutrition [MeSH Terms]) AND Orthopedic) AND outcomes.

### 2.3. Study Selection

Once the duplicates had been removed, articles were retrieved in full text and evaluated. A manual search of the bibliography of each published study was performed in order to find additional articles that could potentially have been missed. Reviews, systematic reviews, and meta-analyses were also retrieved and evaluated in order to broaden the search. The remaining articles were analyzed by two reviewers (P.G. and V.B.) to exclude studies that did not fulfill the eligibility criteria. The reviewers were not blinded to the authors, year, and journal of publication. Clinical studies eligible for inclusion were categorized by study type, according to the Oxford Centre for Evidence-Based Medicine (www.cebm.net (accessed on 13 July 2022). The following categories were utilized: case report (CR), RCTs and case series (CS), in vivo (Iviv), and in vitro (Ivit). Since a score to evaluate such a heterogeneous cohort of studies could not be found, the minimum quality of the study, hence its inclusion, was discussed in the group. After the first selection by one author, studies were presented to the two senior authors (E.F. and R.P.) who performed a final assessment, which included a discussion to reach consensus in case of disagreement.

### 2.4. Data Collection Process

Two assessors (P.G. and L.M.) independently extracted data from eligible studies using a data extraction form that was predefined according to the protocol. For each study, epidemiological characteristics of participants and assessment of results were educed and analyzed using R software (2020; R Core Team).

### 2.5. Data Extraction

All the included studies that met inclusion criteria were categorized and summarized in tables using Microsoft Excel: study type, year of publication, studied surgery, formula of the nutrition used, number of patients, follow-up, route, feeding protocol, and results. Studies were divided into different categories (orthopedic and traumatic).

## 3. Results

A total of 43 studies were found in the electronic search, covering a time period of twenty years (from 1996 to 2017); of these, six were eligible for inclusion in this review. Two more studies were identified as relevant through the manual search and were included. A total of eight studies were thus included. The study selection process is shown in Figure 1.

### 3.1. Non-Elective Trauma Orthopedics Studies

A total of six studies were classified as traumatic (non-elective) surgery (Table 1). All of them were prospective RCTs. The analyzed traumatic patients and surgical procedures were: non-surgical polytraumatic patients requiring enteral nutrition, emergency thorax and abdominal surgery (celiotomy), and mechanically ventilated children with a severe head injury. All studies investigated results during the hospital stay when the immune-enhanced compound was administered through a nasoduodenal tube or jejunostomy. A synopsis of all the clinical studies with their major findings is reported in Table 2. Table 3 compares nutrition ingredients, while formulas and administration schemes are described in Table 4. Finally, different scoring systems assessing the severity of illness and evaluating the prognosis of each patient enrolled are reported in Table 5.

### 3.2. Elective Orthopedic Studies

A total of two studies from elective surgery were classified as orthopedic (Table 6). One study was prospective, while the other was retrospective. The analyzed orthopedic surgical procedures were elective hip and knee arthroplasties. The follow-up period ranged from 60 to 90 days. The most common indication for surgery was primary osteoarthritis. In both studies, the immune-enhanced compound was administered through oral intake. A synopsis of all the orthopedic studies with their major findings is reported in Table 7. Despite the fact that formulas (in kcal) are not mentioned in the included studies, the volume of formula administered is described in Table 7. Lastly, in Table 8, the nutritional formula ingredients are compared.

## 4. Discussion

Over the last twenty years, the role of nutrition has gained relevance in many surgical fields. Several studies have shown that up to 40–50% of patients present in both surgical and medical departments are malnourished already at admission to the hospital [39,40]. However, malnutrition is associated with increased morbidity and a longer length of hospital stay [41], especially among trauma and elective orthopedic patients [42]. Trauma and orthopedic patients suffer from an intense inflammatory response caused by trauma injury, surgical stress, and associated medical conditions and comorbidities, mainly leading to infectious or even life-threatening complications. Several authors have emphasized the importance of effective enhanced nutrition that could prevent the increased risk of developing acute perioperative complications [43,44,45]. Interestingly, a recent meta-analysis highlighted the impact of preoperative administration of an immunonutrition formula in patients who underwent elective major surgery. A shorter hospitalization as well as reduced rates of infection were described when inspiratory muscle training, smoking and alcohol cessation, psychological therapies, weight loss, and preoperative immunonutrition were performed [46]. Thus, in terms of investing in research, clinicians and laboratory groups selected immunonutrition therapy in trauma and surgical patients, especially during the perioperative period [47,48,49,50]. Encouraging findings about the benefits of immunonutrition have been collected by cancer-related surgery [51], while studies on orthopedic and traumatic patients have been limited to date.

This review adds to the current literature by delineating the role of immunonutrition in trauma non-elective and orthopedic elective settings and highlighting the state of research in the field. A total of eight studies have been selected through a literature search (six related to traumatic patients and two related to elective orthopedic surgery). All studies focused on the role of immunonutrition (nutrition enhanced with polyunsaturated/omega-3 fatty acids, arginine, glutamine, antioxidants, and nucleotides) and showed strong benefits in reducing the length of hospital stays and rates of postoperative infectious complications. The oral supplementation of immunonutrients is the most used protocol in elective orthopedic patients, starting five days before surgery and continuing for an additional five days in the postoperative phase. Enteral nutrition via a nasoduodenal or nasogastric tube or jejunostomy is the most common protocol for immunonutrition among trauma patients. Most of the studies had a follow-up time limited to the hospital stay that was longer among the trauma patients as compared to those who underwent elective orthopedic surgery. Additionally, in trauma patients receiving immunonutrition, the authors showed the beneficial effects on time spent in an intensive care unit and the risk of infectious complications. 

In both scenarios, the authors suggested that the beneficial effects of immunonutrition may be related to their modulating effect on the immune system and the inflammatory response associated with trauma and/or surgical stressors [52], then leading to a shorter hospital stay in more recent articles, lower infectious complications, and lower hospital costs [31], while just certain studies investigated additional laboratory or clinical features related to the specific type of surgery [12,13,37,38].

Furthermore, previous clinical studies have already described how the administration of arginine improved bacterial clearance, T-cell function, and wound healing. In this context, the synthesis of nitric oxide from L-arginine is probably responsible for the overall vasodilator effect and the subsequent increase in splanchnic organ perfusion. An excessive systemic inflammatory response after trauma can cause adverse effects due to a change in protein synthesis and proinflammatory cytokine production, including tumor necrosis factor alpha, IL-1β, and IL-6. Inflammation leads, in turn, to a negative nitrogen balance with a subsequent increased rate of catabolic functions and an overall loss of lean mass. As previously shown, N-3 fatty acids modulate the synthesis of different eicosanoids by reducing leukotriene, thromboxane, and prostaglandin levels, which have proinflammatory, immunosuppressive, and vasoconstrictive actions [11,25,53]. The ribonucleotides are indispensable substrates for cells, especially those with rapid turnover. In cases of metabolic stress, an extra supply of nucleotides is necessary to promote adequate proliferation of T lymphocytes [54]. Similar promising results for immunonutrition in surgical patients were highlighted in several fields, such as in patients undergoing surgical treatment for digestive cancers [9] or in patients with HPV-related cervical cancers, where the immune response plays an essential role [9]. In a cardiovascular setting, immunonutrition seems to help ameliorate atheromatic plaque at the inflammatory level, supporting its anti-inflammatory effect [55].

Between orthopedic and traumatic patients, immunonutrition administration results in an effective improvement of the inflammatory condition, reducing the overall rate of complications, both infectious [10,30,31] and not infectious [30], and hospitalization [10,12,29,30,31]. The authors of [31] described fewer intra-abdominal abscesses and major septic complications when a supplemented diet was administered, reflecting a significant reduction in therapeutic antibiotic usage [31]. The overuse or misuse of these medications has been linked to the emergence and spread of microorganisms that are resistant to them, rendering treatment ineffective and increasing hospital costs. According to these results, previous studies showed how in frail subjects, such as polytraumatized or elderly patients with femoral neck fracture, clinical outcomes and postoperative mortality could be improved by daily nutritional supplementation [56]. It has also been proven that malnutrition and inadequate intake of essential and non-essential amino acids increase the risk of bone fractures and the development of non-union as a result of poor bone callus formation, confirming the hypothesis that nutritional supplementation appears to improve outcomes for both trauma and elective patients [57,58]. Moreover, focusing on serum inflammatory biomarkers such as albumin levels <3.5 g/dL or a total lymphocyte count <1.50 cells/mm were correlated with longer hospitalization in patients undergoing total hip arthroplasty or hemiarthroplasty [59].

However, we also highlight some limitations of the presented results. The quality of the studies is poor since most of them are not randomized or even retrospective, the sample size is very small with the exception of one large retrospective study; there is great heterogeneity due to different kinds of traumas; participants’ age range is highly heterogeneous from children to older adults with poor lab characterization, especially in the emergency trauma setting, and the length of follow-ups is mainly limited to hospital stays, making it difficult to conclude survival or quality of life.

Additionally, patients with autoimmune end-stage joint arthritis, if included for elective orthopedic surgery, might not benefit from an immune-enhancing diet.

Even if the evidence is still low to draw reliable conclusions about the efficacy of immunonutrition in the elective orthopedic and traumatic setting, we certainly underline the potential for an immune-enhancing diet in this setting. This review underlies a relevant reduction in the length of a hospital stay, infectious complications, and hospital costs in orthopedic or traumatic patients fed with immune nutrients during their hospitalization. Nevertheless, further research and higher-quality studies should be carried out to evaluate the application of immunonutrition in this field.

## Figures and Tables

**Table 1:** Non-elective traumatic studies.

Study	Year	Case Formula	Control Formula	Type of Study	Trauma/Surgery	Number of Patients
Kudsk et al. [31]	1996	[IED]Immun-Aid, McGaw, Inc., Irvine, CA	Promote [Ross Laboratories, Columbus, OH] and Casec [Mead-Johnson Nutritionals, Evansville, IN]	Prospective	Emergency abdominal surgery (celiotomy)	35
Weimann et al. [12]	1998	IMPACT, Sandoz Nutrition, Beme, Switzerland	Sandoz Nutrition, Beme, Switzerland	Prospective	Emergency thorax and abdominal surgery (celiotomy)	32
Bastian et al. [36]	1998	Sandoz Nutrition, Berna	Unspecified isocaloric and isonitrogenic	Prospective	No-surgery/emergency thorax and abdominal surgery (celiotomy)	29
Briassoulis et al. [37]	2006	Stresson (N.V.NUTRICIA, Zoetermeer, The Netherlands)	Tentrini (N.V.NUTRICIA)	Prospective	Mechanically ventilated children with severe head injury	40
Lorenz et al. [10]	2015	Not specified enteral parenteral isocaloric nutrition with glutamine supplementation	Not specified	Prospective	Extensive ear, nose, and throat tumor surgery and multiple-trauma patients	22
Rai et al. [38]	2017	Neomune	Fresubin HP energy	Prospective	Moderate to severe head injuries requiring enteral nutrition. Surgical and non-surgical patients	36

**Table 2:** Traumatic study rates and specifics.

Study	Follow-Up	Route	Feeding Protocol	Results
Kudsk et al. [31]	Hospital stay	Jejunostomy	200 mL/die.	Hospital stay, infectious complications, hospital costs, and antibiotic usage were significantly higher in the group not fed with an immune-enhanced diet.
Weimann et al. [12]	Hospital stay	Nasoduodenal tube or jejunostomy	A quantity of 25 mL/h for 18 h per die, stopping for the night between 12:00 PM and 6:00 AM. The feeding rate increased by 25 mL/h from day to day, up to a final rate of 150 mL/h. Simultaneously with enteral nutrition, patients were also fed parenterally until complete enteral coverage of the caloric requirements of 35–40 kcal per kg.	Significantly fewer days of Systemic Inflammatory Response Syndrome (SIRS) per patient and lower scores for the Multiple Organ Failure (MOF) score were observed; outcome and hospital stay were not influenced.
Bastian et al. [36]	Hospital stay	Nasoduodenal tube or jejunostomy	561 ± 266 mL mean/die.	Significant reduction of Systemic Inflammatory Response Syndrome (SIRS), no impact on mortality, intensive care unit, or hospital stay.
Briassoulis et al. [37]	Hospital stay	Nasogastric tube	Hourly amount protocol for the children: energy intake equal to 0.50%, 100%, 125%, 150%, and 150% of the Predicted Basal Metabolic Rate (PBMR) on days 1–5, respectively. Enteral feeding was increased during the first five days, eventually reaching 150% of the PBMR.	Significantly lower interleukin-8 levels and positive gastric cultures. No differences were found in nosocomial infections, length of stay, length of mechanical ventilation, or survival.
Lorenz et al. [10]	Hospital stay	Enteral not specified	Not specified.	Decreased septic complications, length of hospital stays, length of intensive care unit, and time for wound healing. A small cohort of patients and little data reported.
Rai et al. [38]	Six months	Nasogastric tube	The target calorie for each patient was determined by the clinician working alongside the dietitian using the Harris–Benedict equation, which measures resting energy expenditure. Enteral feeding was commenced at an initial rate of 20 mL/h and increased by 20 mL/h every 6 h until the target calorie was reached.	IL-6 levels were significantly reduced, whereas glutathione levels were significantly higher following feeding with immunonutrition. Only inflammatory blood curves are analyzed, not clinical or economic benefits.

**Table 3:** Immunonutrition formula ingredients.

Study	Formula	Control
Kudsk et al. [31]	Water, Corn Maltodextrin, Sodium Caseinate, Soy Protein Isolate, Soy Oil, Sugar. Less than 0.5% of Medium Chain Triglycerides, Safflower Oil, Natural and Artificial Flavors, Calcium Citrate, Potassium Chloride, Magnesium Phosphate, Potassium Citrate, Calcium Phosphate, Soy Lecithin, Choline Chloride, Ascorbic Acid, Taurine, l-Carnitine, DL Alpha-Tocopheryl Acetate, Ferrous Sulfate, Zinc Sulfate, Manganese Sulfate, Niacinamide, Calcium Pantothenate, Copper Sulfate, Riboflavin, Thiamine Hydrochloride, Pyridoxine Hydrochloride, Vitamin A Palmitate, Beta-Carotene, Chromium Chloride, Folic Acid, Potassium Iodide, Sodium Molybdate, Sodium Selenate, Phylloquinone, Biotin, Vitamin D3, and Vitamin B12	Water, Corn Maltodextrin, Sodium Caseinate, Soy Protein Isolate, Soy Oil, and Sugar
Weimann et al. [12]	Water, Sugar, Calcium Caseinate (Milk), Sodium Caseinate, l-Arginine, Refined Fish Oil (anchovy and sardine) and less than 2% of Corn Oil, Citric Acid, Medium Chain Triglycerides, Maltodextrin, Natural and Artificial Flavor, Yeast Extract, Potassium Citrate, Potassium Chloride, Calcium Phosphate, Magnesium Phosphate, Salt, Magnesium Chloride, Cellulose Gel, Cellulose Gum, Choline Chloride, Sodium Ascorbate, Sucralose (Sweetener), Carrageenan, Zinc Sulfate, Ferrous Sulfate, D Alpha-Tocopherol, Niacinamide, Soybean Oil, DL Alpha-Tocopheryl Acetate, Calcium Pantothenate, Copper Gluconate, Manganese Sulfate, Vitamin A Palmitate, Pyridoxine Hydrochloride, Riboflavin, Thiamine Hydrochloride, Beta-Carotene, Folic Acid, Potassium Iodide, Sodium Selenite, Sodium Molybdate, Chromium Chloride, Vitamin K1, Biotin, Vitamin D3, and Vitamin B1	Sugar, Whey (Milk) Protein, Maltodextrin, l-Arginine, Fish Oil, Minerals (Potassium Dihydrogen Phosphate, Ferrous Sulphate, Zinc Sulfate, Potassium Iodide, Copper Gluconate, Chromium Trichloride, Manganese Sulfate, Sodium Selenite, Sodium Molybdate, and Sodium Fluoride), Partially hydrolyzed Guar gum fire, Medium Chain Triglycerides Oil, Corn Oil, Acidity regulator (Citric Acid), Flavors (Mango, Grapefruit, and Passion fruit), Ribonucleic Acid Sodium Salt, Vitamins (C, E, B5, A, D, Niacin, B6, B1, B2, K, Folic Acid, and d-Biotin), Stabilizer (Xanthan gum), Choline Bitartrate, Emulsifier (Soy Lecithin), Colors (Beta Carotene and Beetroot Red), Antioxidants (l-Ascorbyl Palmitate and Tocopherol Rich-extract). Gluten free
Bastian et al. [36]	Water, Sugar, Calcium Caseinate (Milk), Sodium Caseinate, l-Arginine, Refined Fish Oil (anchovy and sardine) and less than 2% of Corn Oil, Citric Acid, Medium Chain Triglycerides, Maltodextrin, Natural and Artificial Flavor, Yeast Extract, Potassium Citrate, Potassium Chloride, Calcium Phosphate, Magnesium Phosphate, Salt, Magnesium Chloride, Cellulose Gel, Cellulose Gum, Choline Chloride, Sodium Ascorbate, Sucralose (Sweetener), Carrageenan, Zinc Sulfate, Ferrous Sulfate, D Alpha-Tocopherol, Niacinamide, Soybean Oil, DL Alpha-Tocopheryl Acetate, Calcium Pantothenate, Copper Gluconate, Manganese Sulfate, Vitamin A Palmitate, Pyridoxine Hydrochloride, Riboflavin, Thiamine Hydrochloride, Beta-Carotene, Folic Acid, Potassium Iodide, Sodium Selenite, Sodium Molybdate, Chromium Chloride, Vitamin K1, Biotin, Vitamin D3, and Vitamin B12	Not specified
Briassoulis et al. [37]	Nitrogen, Casein l-Arginine, l-Glutamine, Sugar, Lactose, Polysaccharides, Saturates, Mono unsaturates, Polyunsaturated Linoleic acid, Alpha-Linolenic acid, Docosahexaenoic acid, Eicosapentaenoic acid, Sodium, Potassium, Calcium, Phosphate, Magnesium, Vitamin E, Carotenoids, Taurine, Carnitine, Selenium, Zinc, and Copper	Water, Maltodextrin, Fructose, Potassium Citrate, Sodium Citrate, Acidity Regulator (Citric Acid), Flavoring, and Sweeteners (Acesulfame K and Sodium Saccharin)
Lorenz et al. [10]	Not specified: enteral parenteral isocaloric nutrition with glutamine supplementation	Not specified
Rai et al. [38]	Maltodextrin, Sodium Caseinate, Medium chain Triglycerides Oil, Dry Omega-3, Arginine, Fructose, Poly Dextrose, Corn Oil, Glutamine, Citric Acid Anhydrous (Acidity Regulator), Trehalose (Sweetener), Potassium Chloride, Dicalcium Phosphate 2-hydrate, Choline Bitartrate, Mono- and Diglycerides of Fatty Acids (Emulsifier), Calcium Carbonate, Beta-Carotene 1%, Magnesium Oxide, Potassium Acetate, Sodium Ascorbate (Vitamin C), De-oiled Enzyme-Modified Soy Lecithin (Emulsifier), DL Alpha-Tocopherol Acetate (Vitamin E-Acetate 50%), Sodium Ferrous Citrate, Calcium Chloride Dihydrate, Taurine, Carnitine, Zinc Sulfate Heptahydrate, Biotin 1%, Niacin, Vitamin B12 0.1%, Dry Vitamin A (175,000 IU/g), Vitamin E (Mixed Tocopherols Concentrate) (Antioxidant), Calcium Pantothenate (Vitamin B5), Manganese Sulfate Monohydrate, Copper Sulfate Pentahydrate, Thiamine HCl (Vitamin B1), Pyridoxine HCl (Vitamin B6), Selenium Glycinate Complex 1.0%, Molybdenum Glycinate Chelate 2.5%, Chromium Nicotinate Glycinate Chelate 2.5%, Riboflavin (Vitamin B2), Dry Vitamin D3 (200,000 IU/g), Folic Acid (Vitamin M), Potassium Iodide, and Vitamin K1 powder 50%	Water, Maltodextrin, Milk protein, Medium chain Triglycerides, Vegetable Oils (Soya Oil and Linseed Oil), Potassium Citrate, Fish Oil, Potassium Chloride, Sodium Chloride, Calcium Chloride, Sodium Citrate, Vitamin C, Choline Chloride, Magnesium Oxide, Calcium Phosphate, Acidity Regulator (E330), Magnesium Citrate, Emulsifier (E471), Zinc Sulphate, iron Pyrophosphate, Iron Sulphate, Niacin, Vitamin E, Manganese Chloride, Pantothenic Acid, Sodium Fluoride, Vitamin B2, Copper Sulphate, Vitamin B6, Vitamin B1, Vitamin A, Beta-Carotene, Folic Acid, Sodium Molybdate, Potassium Iodide, Chromium Chloride, Sodium Selenite, Vitamin K1, Biotin, Vitamin D3, and Vitamin B12

**Table 4:** Immunonutrition formula kilocalories and volume administered.

Study	kcal	Volume
Kudsk et al. [31]	Immun-Aid = 1040 kcal Promote and Casec = 1000 kcal	Not specified
Weimann et al. [12]	IMPACT Sandoz Nutrition = 101 kcal × 100 mL Sandoz Nutrition = 101 kcal × 100 mL	35 ± 40 kcal/kg body weight/day
Bastian et al. [36]	IMPACT Sandoz Nutrition = 101 kcal × 100 mL Not specified control = 101 kcal × 100 mL	35 ± 40 kcal/kg body weight/day
Briassoulis et al. [37]	Stresson = 100 kcal × 100 mL Tentrini = 125 kcal × 100 mL	According to the predicted basal metabolic rate
Lorenz et al. [10]	Not specified	
Rai et al. [38]	Neomune = 1503 kcal Fresubin^®^ HP energy = 1502 kcal	About 1000 mL in both groups

**Table 5:** Assessment of the patient’s clinical situation. Injury Severity Score (ISS), ATI Score (age-thrombus burden index of microcirculatory resistance), Glasgow Coma Scale (GCS), APACHE II (Acute Physiologic Assessment and Chronic Health Evaluation II), Pediatric Trauma Score (PTS), PRISM (Pediatric Risk of Mortality), and Therapeutic Intervention Scoring System (TISS).

Study	Scoring System
Kudsk et al. [31]	ISS ≥ 21 ATI ≥ 25 GCS ≥ 7
Weimann et al. [12]	ISS-test: 39.6 ± 11.4 ISS-control: 40.5 ± 9.2 APACHE II-test: 6.5 ± 3.1 APACHE II-control: 9.8 ± 6.3
Bastian et al. [36]	ISS-test: 39.6 ± 11.4 ISS-control: 40.5 ± 9.2 APACHE II-test: 6.5 ± 3.1 APACHE II-control: 9.8 ± 6.3 PTS-test: 38.8 ± 12.5 PTS-control: 40.8 ± 15.5
Briassoulis et al. [37]	PRISM: 13.2 ± 1 PRISM-control: 12.4 ± 1 TISS: 32.5 ± 4 TISS-control: 28.4 ± 3.7 GCS: 6.1 ± 0.4 GCS-control: 6.3 ± 0.6
Lorenz et al. [10]	ISS, GCS, APACHE 11, MOF, SOFA, and SAPS values not cited
Rai et al. [38]	GCS from moderate to severe (3–12)

**Table 6:** Elective orthopedics studies.

Study	Year	Formula	Control Formula	Type of Study	Surgery	Number of Patients	Age	Sex
Alito et al. [29]	2016	ACERTO protocol	Impact-Nestlé, Brazil	Prospective Pilot	Elective total hip arthroplasty	32	58 years; range, 26–85 years	50% women
Gonçalves et al. [30]	2021	ONS with impact immunonutrients (Nestlé Health Science, Epalinges, Switzerland)	Not specified	Retrospective cohort	Elective total hip and knee arthroplasties in elderly patients	3015	72.6 ± 6.9 years	81.2% women

**Table 7:** Elective orthopedic study rates and specifics.

Study	Follow-Up	Route	Feeding Protocol	Results
Alito et al. [29]	Hospital stay	Oral intake	600 mL per day	Reduction in length of hospital stay and postoperative acute inflammatory response.
Gonçalves et al. [30]	3 months	Oral intake	600 mL per day	Shorter length of hospital stay and a lower rate of infectious complications compared to the control group. A 55% reduction in the chance of infectious complications and mean hospitalization was 42% lower in the immunonutrition group. Additionally, the immunonutrition group reduced the chance of secondary outcomes, such as non-infectious complications and the need for blood transfusion, by 50–76% in an adjusted logistic regression model, respectively.

**Table 8:** Immunonutrition formula ingredients.

Study	Formula	Control
Alito et al. [29]	Proteins 23% (77% Calcium Caseinate and 23% arginine); Carbohydrates 52% (100% Maltodextrin); Lipids 25% (68% Fish Oil, 20% Medium-chain Triglycerides, and 12% Corn Oil); Vitamins and Electrolytes	Water, Maltodextrin, Milk Proteins, Vegetable Oils (Palm and Sunflower), Minerals (Potassium Citrate, Calcium Chloride, Magnesium Citrate, Trisodium Citrate, Potassium Phosphate, Sodium Chloride, Potassium Chloride, Zinc Sulphate, Ferrous Sulphate, Copper Gluconate, Manganese Sulphate, Sodium Fluoride, Sodium Molybdate, Chromium Chloride, Sodium Selenite, and Potassium Iodide), l-Arginine, Fish Oil, Yeast Extract Rich In Nucleotides (RNA), Emulsifier (Soy Lecithin), Choline Bitartrate, Vitamins (C, E, Pantothenic Acid, Niacin, B1, B6, B2, A, D, B12, K, Folic Acid, and Biotin), Acidity Regulator (E330), Antioxidants (E301 and E307), and Color (Beta-Carotene)
Gonçalves et al. [30]	Water, Sugar, Calcium Caseinate (Milk), Sodium Caseinate, l-arginine, Refined Fish Oil (Anchovy and Sardine) and less than 2% of Corn Oil, Citric Acid, Medium-chain Triglycerides, Maltodextrin, Natural and Artificial Flavor, Yeast Extract, Potassium Citrate, Potassium Chloride, Calcium Phosphate, Magnesium Phosphate, Salt, Magnesium Chloride, Cellulose Gel, Cellulose Gum, Choline Chloride, Sodium Ascorbate, Sucralose (Sweetener), Carrageenan, Zinc Sulfate, Ferrous Sulfate, D Alpha-Tocopherol, Niacinamide, Soybean Oil, DL Alpha-Tocopheryl Acetate, Calcium Pantothenate, Copper Gluconate, Manganese Sulfate, Vitamin A Palmitate, Pyridoxine Hydrochloride, Riboflavin, Thiamine Hydrochloride, Beta-Carotene, Folic Acid, Potassium Iodide, Sodium Selenite, Sodium Molybdate, Chromium Chloride, Vitamin K1, Biotin, Vitamin D3, and Vitamin B12	Not specified

## Data Availability

Not applicable.
